# Supplementary material for: The Systems Biology Research Tool: evolvable open-source software
Source: BMC Syst Biol. 2008 Jun 29;2:55. doi: 10.1186/1752-0509-2-55 (PMC2446383; doi:10.1186/1752-0509-2-55)
Supplement: Additional file 1 — SBRT Archive. An archive of the current version of the Systems Biology Research Tool. [file 1752-0509-2-55-S1.zip › sbrt-1.4.0/doc/developers_guide/api/sbrt/kernel/io/SingleRm.html]

SingleRm


|  |  |  |
| --- | --- | --- |
| **Overview** \| **Package** \| **Class** \| **Use** \| **Tree** \| **Deprecated** \| **Index** \| **Help** | |  |
| **PREV CLASS**   **NEXT CLASS** | **FRAMES**    **NO FRAMES**     **All Classes** |  |
| SUMMARY: NESTED \| FIELD \| CONSTR \| METHOD | DETAIL: FIELD \| CONSTR \| METHOD |  |


---


## sbrt.kernel.io Class SingleRm<R>

```
java.lang.Object
  sbrt.kernel.io.AbstractResultsManager<R>
      sbrt.kernel.io.SingleRm<R>
```

**Type Parameters:**: `R` - the result type.

**All Implemented Interfaces:**: ResultsManager<R>

---

``` public class SingleRm<R> extends AbstractResultsManager<R> ```

This class is used to store a single result that can be easily retrieved.

**Author:**
:   This class was written and documented by Jeremiah Wright
    while in the Wagner lab.

---

**Constructor Summary**

`SingleRm()`


| **Method Summary** | |
| --- | --- |
| `<S extends R> void` | `addData(S results)`             Adds the provided results to this results manager. |
| `R` | `getResults()`             Returns the last result added to this results manager. |

**Methods inherited from class sbrt.kernel.io.AbstractResultsManager**

`breakPoint, checkState, close, getName, isClosed`

**Methods inherited from class java.lang.Object**

`clone, equals, finalize, getClass, hashCode, notify, notifyAll, toString, wait, wait, wait`

**Constructor Detail**

### SingleRm

```
public SingleRm()
```


**Method Detail**

### getResults

```
public R getResults()
```

:   Returns the last result added to this results manager.

    :   **Returns:**: the last result added to this results manager.

---


### addData

```
public <S extends R> void addData(S results)
```

:   Adds the provided results to this results manager. If a previous
    result exists, it will be discarded and replaced with the result
    provided here.

    :   **Type Parameters:**: `S` - a type extending the primary result type. **Parameters:**: `results` - the results to be stored.


---


|  |  |  |
| --- | --- | --- |
| **Overview** \| **Package** \| **Class** \| **Use** \| **Tree** \| **Deprecated** \| **Index** \| **Help** | |  |
| **PREV CLASS**   **NEXT CLASS** | **FRAMES**    **NO FRAMES**     **All Classes** |  |
| SUMMARY: NESTED \| FIELD \| CONSTR \| METHOD | DETAIL: FIELD \| CONSTR \| METHOD |  |


---
